# Development of 60-GHz millimeter wave, electromagnetic bandgap ground planes for multiple-input multiple-output antenna applications

**DOI:** 10.1038/s41598-020-65622-9

**Published:** 2020-05-22

**Authors:** Sana Ullah, Woon-Hong Yeo, Hongjoon Kim, Hyoungsuk Yoo

**Affiliations:** 1 Department of Biomedical Engineering, Hanyang University, Seoul, 04763 Republic of Korea; 2 George W. Woodruff School of Mechanical Engineering and Wallace H. Coulter Department of BiomedicalEngineering, Georgia Institute of Technology, Atlanta, GA 30332 USA; 3 Department of Electrical Engineering, Kyungpook National University, Daegu, 41566 Republic of Korea

**Keywords:** Electrical and electronic engineering, Metamaterials

## Abstract

For 60-GHz band communications, both the mutual coupling and transmission distance restrict the performance of a multiple-input multiple-output (MIMO) antenna array. Several studies presented different types of meta-materials and electromagnetic bandgap (EBG) structures to improve the performance of a MIMO antenna array at the 60-GHz band. In this paper, we presented the four-element MIMO patch antenna with different types of EBG structures for the millimeter wave (mmW)communications at the 60-GHz unlicensed industrial, scientific, and medical band. The single element of the MIMO antenna array covered the mmW band from 57 GHz to 63 GHz having the dimensions of 1.3 mm × 1.8 mm × 0.1 mm. We developed a set of square-shaped, cross-shaped, and complex-slotted EBG ground planes between the antenna elements for the performance improvement. All the three EBG ground planes provided significant coupling reduction between the mmW MIMO antenna elements. The proposed EBG structures exhibited wide bandgap characteristics and improved scattering parameters in the desired frequency band. In contrast with the cross- and complex-slotted, the square-shaped EBG structure substantially improved the overall gain of MIMO antenna array. In addition, the square-shaped EBG reformed the maximum beam and enhanced the far-field gain pattern in the desired direction. Experimental results conducted with the fabricated prototypes showed a good agreement with the simulation results and adequately covered the 60-GHz band. The low-profile and salient features of the proposed MIMO antenna array shows the potential for on-chip applications at 60 GHz.

## Introduction

Broadband systems are becoming more ubiquitous, and at the same time, wireless technology is rapidly spreading. Over the previous decades, the array antenna systems have been used for the gain, efficiency, and multiple-input multiple-output (MIMO) applications for 4G and 5G wireless communication network devices^1^. In^2^, used a MIMO antenna for miniaturization of the array system in the wearable application. In a MIMO system, multiple antenna elements are used for high data rates by increasing the channel capacity^3,4^. Hassan and Sharawi^5^ presented four half circle-shaped printed monopole antennas for MIMO applications to provide a measured efficiency and gain of 81.5% and 3.12 dBi, respectively. However, for 5G wireless communication, the millimeter-wave (mmW) frequency band from 57 to 64 GHz is a promising solution. At mmW band peak absorption due to water and oxygen molecules at the atmospheric limit the propagation of waves, and the transmission distance becomes less than 1 kilometer^6^. Therefore, mmW band provides the frequency reuse for MIMO networks and allows short-range ethernet bridges^7^. The small range communication in the 60 GHz required a multi Gb/s data transmission; therefore, a photonics-based mmW antenna was utilized for high-speed data transmission^8^. At mmW two Fresnel-zone plate antennas with the pure plastic material were used to achieve a high efficiency over the considered operating band^9^. The currently deployed wireless communication systems are overcrowded, which make the mmW band suitable for communications.

Most of the performance metrics of a MIMO system rely on the overall MIMO element’s gain and the coupling between ports. Therefore, the coupling between closely spaced antennas at the mmW or 60 GHz band was reduced using meta-materials or electromagnetic bandgaps (EBG)^10–14^. However, all the aforementioned metamaterials were placed in a vertical position between two antennas due to the dielectric resonator antenna (DRA), which makes the metamaterials placement slightly complex in MIMO antenna arrays. The extra circuit elements or meta-materials were used with the antennas to facilitate stable resonant frequencies and enhanced efficiency^15,16^, but the metamaterials can work only at the lower frequency bands (2.4 GHz and 400 MHz). A simple and compact circuit-decoupling technique using two transmission lines and a shunt reactive component followed by an impedance-matching network were used for approximately 20 dB coupling reduction and 3 dB gain improvement^17^. The work presented in^18^, used an mmW DRA surrounded by an EBG structure for the 60-GHz frequency to achieve gain improvement up to 3.2 dB; however, the EBG structure was simple (circular-shaped) and used only with a single antenna. An EBG reflector for the 60-GHz application provided low backward radiation and increased the bandwidth of a square-slot antenna array^19^, but the target antenna array was a simple array rather than a MIMO antenna array. Moreover, different types of EBG structures were integrated with a single antenna and with a 2 × 2 patch antenna array to suppress or absorb the electromagnetic waves and to enhance the broadside directivity and gain over the 60-GHz band^20,21^, though the study used a simple EBG structure (mushroom EBG) and a simple patch antenna array. In^22^, the authors suggested an EBG based on the photonic bandgap (PBG) phenomenon using periodic structures. The EBG can be used to enhance the antenna performance, such as the directivity, gain, and multi-band operations^23^. Mu’ath *et al*.^24^ used a DRA with EBG materials for the coupling reduction. The authors implemented a novel shaped complex-slotted EBG without a vertical component to provide a drastic decrease in mutual coupling at the 60-GHz band. In^25^, coupling reduction in a microstrip patch antenna array was achieved using parallel coupled-line resonators with an enhancement of the antenna gain up to 1.25 dB. A uni-planar compact EBG at 60 GHz was simulated and fabricated over a low-temperature cofired-ceramic substrate to increase the antenna gain (2.3 dBi) and reduce the mutual coupling (11.8 dB) in a simple patch-antenna 16-element array^26^. The aforementioned studies used either DRA or a simple patch antenna array with simple shape EBG structures or unique complex structures, and most of the aforementioned studies used the simple antenna array not the MIMO antenna array. Several researchers proposed different types of EBG structures at the 60 GHz band; however, less attention has been paid to the performance comparison of the EBGs at the 60 GHz band based on the shape complexity. At the 60 GHz band, the shape of the EBG structure and its implementation with MIMO antenna array is an important factor in terms of the MIMO performance.

In this paper, we proposed a 60-GHz mmW four-element edge-fed MIMO patch-antenna array with a simple shape EBG structure. We designed a set of square-shaped, cross-shaped, and complex-slotted^24^ structure of the EBG ground planes and compared the performance at the 60-GHz frequency band in terms of the coupling reduction, bandgaps, and gain enhancement. Different simulations were conducted using a high frequency structural simulator (HFSS) software from ANSYS, Inc. We selected the EBG unit cell dimensions based on the transmission and reflection coefficients for the surface wave suppression at 60 GHz band. The rest of the paper is organized as follows. Section 2 briefly describes the characteristics of the 60-GHz four-element MIMO antenna. The design specifications of the different EBG structures and the results and discussion are presented in Sections 3 and 4, respectively. Finally, the paper concludes with the main results in Section 5.

## MIMO antenna array Design Details

**Figure 1:**
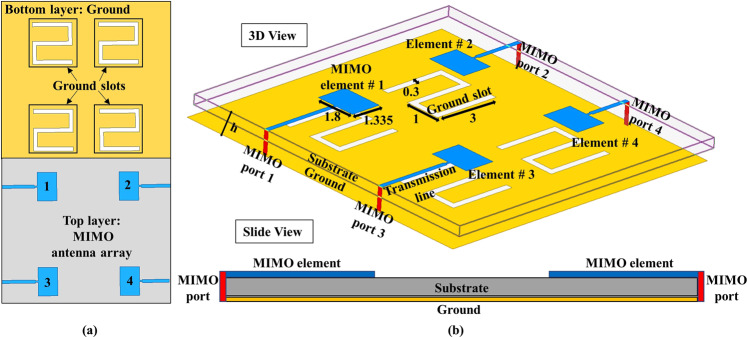
Geometry of the proposed four-elements MIMO antenna (Units: millimeters) (**a**) front view and (**b**) 3D and side view. The MIMO antenna is consisted of three layers. The top, middle, and bottom layers contained the antenna elements, substrate, and a ground plane, respectively.

In a MIMO antenna system, multiple elements are used to improve the gain or increase the channel capacity. MIMO applications need more antenna elements for the transmission and reception of signals to avoid the fading loss. Therefore, we proposed a four-element 60-GHz MIMO antenna array system for the mmW MIMO applications. The layer and three-dimensional views of the proposed MIMO antenna with their dimensions are shown in Fig. 1(a,b), respectively. To avoid the fabrication complexities, we opt to design a simple and conventional edge-fed patch antenna. For the array, four elements of the patch antenna are proposed. As shown in the figure, the top layer consists of the main radiator for each element and four edge-fed ports located at the end of each transmission line. A Z-shaped slots in the ground plane are introduced to achieve a better impedance matching and tuning of the proposed MIMO antenna at the desired operating band, as depicted in Fig. 1(a). The antenna is mounted on a Rogers ULTRALAM substrate with permittivity (*ε*_r_) of 2.9, which is easily available at a low price. The dimensions of a single element are 1.335 mm × 1.8 mm × 0.1 mm, whereas the overall dimensions of the MIMO antenna array are 13 mm × 14 mm × 0.1 mm.

### Effects of the Z-shaped ground slot

**Figure 2:**
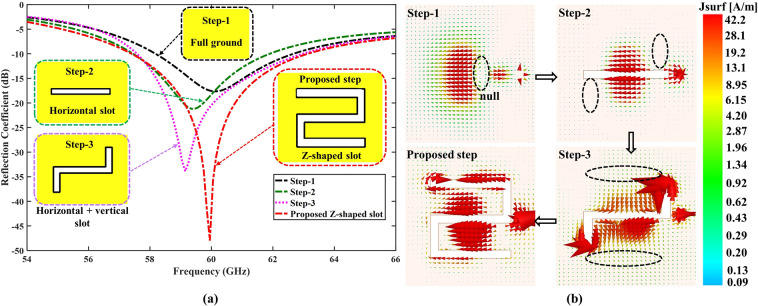
Effects of the ground plane slot on the *S*_11_ performance of the antenna. (**a**) Comparison of the scattering parameter (*S*_11_) and (**b**) current distributions on the ground slot.

Figure 2 illustrates the comparison of the scattering parameter (*S*_11_) for the involved steps in the design of the ground plane. Initially, we want the antenna to be as simple as possible and start from a typical full ground plane shown as step-1. However, the antenna operates in a 60.7 GHz frequency with narrow bandwidth and a weak resonance mode at 60 GHz range due to unbalanced currents at the ground plane as shown in Fig. 2(b). Therefore, to tune and match the antenna at the desired 60 GHz band without increasing the size of the antenna, we introduced a horizontal slot in the ground (step-2), which shifted the resonance frequency slightly to lower and increased the resonance depth (*S*_11_ <−20 dB) at 59.5 GHz. By inserting two vertical slots at both ends of the horizontal slot (step-3), this lengthens the current path on the ground than that in step-2, which shifted the frequency band to 59.2 GHz with slightly wider bandwidth and increased the resonance depth (*S*_11_ <−33 dB). From step-3, we achieved the wider bandwidth, increased resonance depth, and lengthen current path, however, the resonance mode of the antenna was shifted from our desired 60 GHz frequency band. Finally, by inserting a Z-shaped slot in the ground (proposed step), the current path on the ground becomes much longer and more balanced as compared to the previous step (step-3); hence the antenna resonance mode matched at the 60 GHz (*S*_11_ <−47 dB) with wide bandwidth (5.9 GHz). Figure 2(b) shows that the currents distribution on the slotted ground (proposed step) reverses the direction of their flow many times, which improves the impedance matching and bandwidth.

## EBG Design

**Figure 3:**
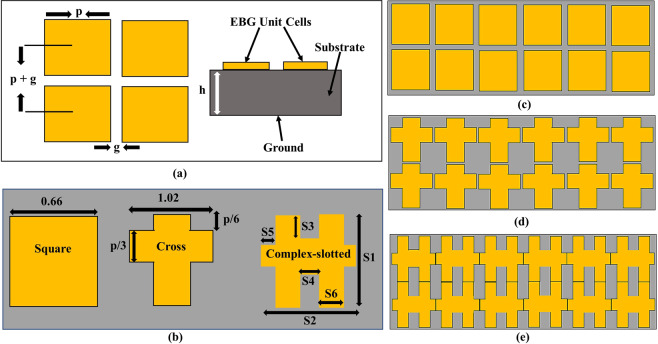
Proposed model for the EBG structures (Units: millimeters) (**a**) mushroom-type EBG structure (top view and side view), (**b**) unit cell of the EBGs, (**c**) array of a square-shaped EBG, (**d**) array of a cross-shaped EBG, and (**e**) array of a complex-slotted EBG. EBG unit cell dimensions are as follows: *p* = 0.66, *g* = 0.3, S_1_ = 0.98, S_2_ = 0.23, S_3_ = 0.39, S_4_ = 0.05, S_5_ = 0.04, and S_6_ = 0.05.

In general, a conventional mushroom-type EBG structure is composed of a ground plane, equal-length patches, and an insulating material such as a dielectric substrate between the patches and the ground, as shown in Fig. 3(a). In the low-profile antennas, it was reported that the mushroom-type EBG structure mimics the properties of a high impedance surface (HIS)^12^. Owing to the surface current propagation characteristics, like HIS, the EBG structure blocks or suppresses the leaky or degradable waves in the operating frequency band. According to the desired application, the EBG structure can be implemented in different types of periodic shapes. The gap between the EBG unit cells should be narrower than the wavelength of the operating frequency. The surface impedance properties of the mushroom-type EBG structure can be extracted from its equivalent LC circuit model. The exact and optimized values of the inductance *L* and capacitance *C* for the desired frequency band are determined using the following equations^27,28^, which are in-turn used to calculate the bandwidth (BW) of the EBG at the desired bandgap.

1 $$L={\mu }_{o}h$$

2 $$C=\frac{P{\varepsilon }_{o}\mathrm{(1}+{\varepsilon }_{o})}{\pi }\cos {h}^{-1}\left(\frac{2P+g}{g}\right)$$

3 $${f}_{r}=\frac{1}{2\pi \sqrt{LC}}$$

4 $$BW=\frac{1}{\eta }\sqrt{\frac{L}{C}}$$

In (1) and (2), *μ*_o_ and *ε*_o_ are the permeability and the permittivity of the free space, respectively; whereas in (3) and (4), *f*_*r*_ and *η* are the angular frequency and the wave impedance of the free space, respectively. The capacitance between the adjacent patches depends on the gap (*g*) variation, whereas the inductance depends on the height (*h*) of the substrate. The EBG unit cell is optimized by the two parameters: patch width (*p*) and *g*. The desired resonant frequency (*f*_*r*_) and *BW* of the bandgap are optimized by the surface capacitance *C* and the inductance *L*. These equations provide an approximation about the frequency bandgap (EBG surface current restriction bandgap). With Eq. (3), we can design a compact EBG by increasing either the capacitance or the inductance. To increase the inductance, we can use an approach used in microwave circuits in which the curves are etched into the EBG metal patches, like a coplanar spiral inductor. For the basic EBG structures, the characteristic wavelength is used to optimize the length and width of the unit cells, as well as the gap between them with Eqs. (5) and (6)^29^:

5 $$P=0.12\lambda $$

6 $$g=0.02\lambda $$

However, with the help of the above-mentioned formulas, complex structures other than the conventional structure are difficult to obtain. Therefore, we used a variety of EBG structures to operate at our desired frequency band. The proposed EBG structures are shown in Fig. 3(b–e). The square-shaped and cross-shaped EBG structures were designed to reduce the coupling between the elements and improve the overall MIMO antenna gain. The dimensions of unit cells for each type of EBG are shown in Fig. 3(b) and given in the caption of the figure, whereas the geometric models of the square- and cross-shaped EBG structures are shown in Fig. 3(c,d), respectively. In Fig. 3(e), the complex-slotted structure is presented. The inductance value of the unit cell was increased by lengthening the connecting bridges^24^. The dimensions of the compact complex-slotted EBG structure were taken from Mu’ath *et al*.^24^ and are summarized in Fig. 3’s caption.

**Figure 4:**
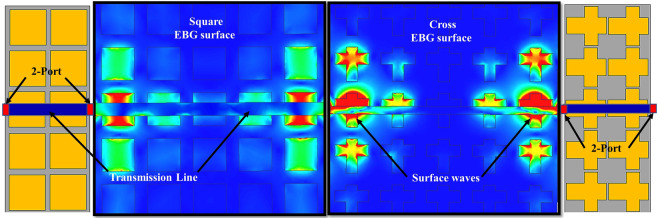
EBG setups with two port transmission lines for the surface wave suppression. The transmission lines were placed 0.3 mm above the square- and cross-shaped EBG structures. The surface waves were suppressed by the EBG structures and were blocked from the propagation through transmission lines.

**Figure 5:**
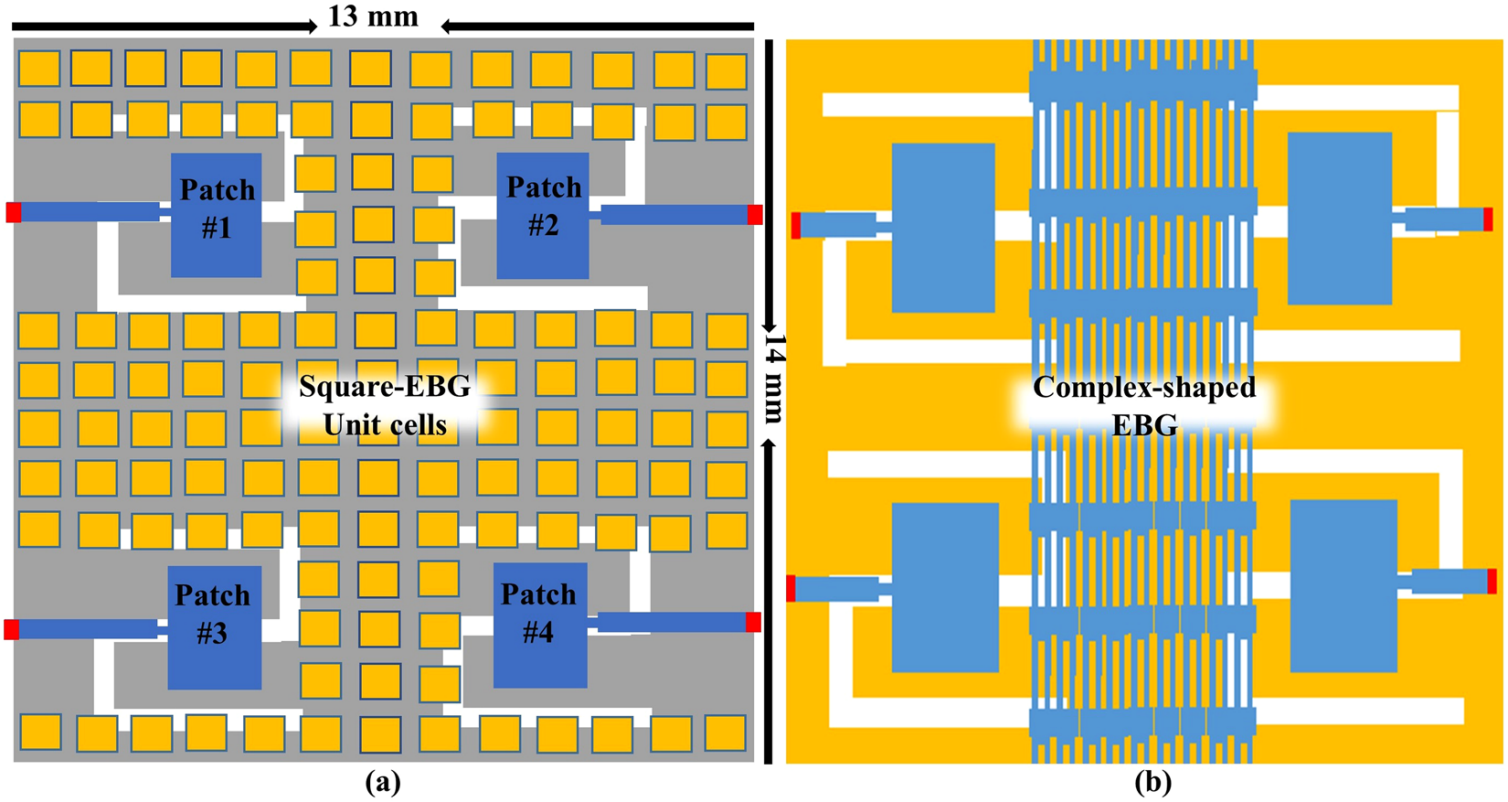
Four-elements MIMO antenna array with EBG structures. (**a**) Square-shaped EBG. (**b**) Complex-slotted EBG. The square-shaped EBG structure was placed all over the MIMO elements, whereas the complex-slotted EBG structure was inserted in the middle of array elements.

To validate the surface wave suppression property of the EBGs structures, a narrow strip of the transmission line was placed on the top of the EBG surface. Both ends of the transmission line were connected to two ports at a distance of 0.3 mm above the EBG surface. The setups of the surface wave suppression for both square- and cross-shaped EBG structures are shown in Fig. 4. After analyzing the surface wave suppression results, the EBG structures were ready to place between the 60-GHz MIMO antenna array for comparison in terms of the mutual coupling and gain improvement. Figure 5 shows the implementation of the square-shaped and complex-slotted EBG structures on the proposed four-element MIMO antenna array. The EBG unit cells are placed at a constant periodicity over the MIMO antenna to provide surface wave suppression. To block the surface current at the resonant frequency, the *g* and slots in the cross-shaped EBG unit cell provides the capacitance and inductance, respectively. The placement of the cross-type EBG structure on the four-element MIMO antenna is the same as of the square-shaped EBG structure. The compact-size complex-slotted EBG structure is placed in such a way that two elements of the array are on the one side and the two elements are on the other side of the structure, as shown in Fig. 5. To verify the simulation results, we fabricated the 60-GHz MIMO antenna array and two of the EBG structures (i.e., square- and cross-shaped) and measured the results with a high-frequency vector network analyzer (VNA).

## Results and Discussions

### MIMO antenna array with and without EBG

**Figure 6:**
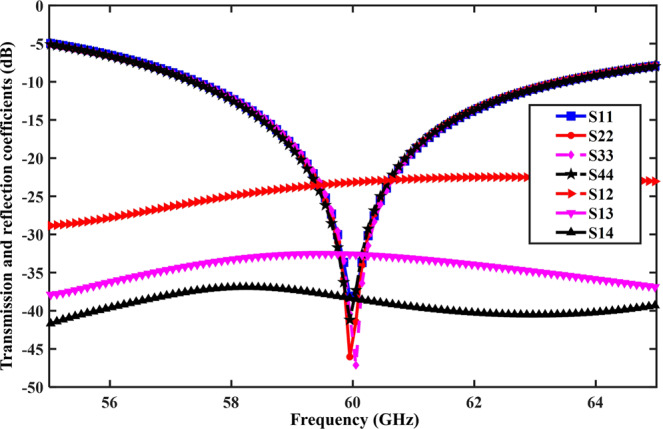
Antenna characteristics: reflection coefficients (*S*_11_, *S*_22_, *S*_33_, and *S*_44_) and transmission coefficients (*S*_12_, *S*_13_, and *S*_14_) of the four-element MIMO antenna array. The scattering parameters were matched exactly at the desired 60-GHz band.

Figure 6 shows the reflection coefficients (*S*_11_, *S*_22_, *S*_33_, and *S*_44_) of the proposed MIMO antenna, which cover the unlicensed band at 60 GHz (57–63 GHz) with a relatively high gain of 8.2 dBi. It is clear from the figure that the reflection coefficient of each antenna element coincides with the antenna working frequency (60 GHz) and provides a better impedance matching of −45 dB. The simulation results of the transmission coefficient (*S*_12_) between the elements 1 and 2 is also presented in Fig. 6. The transmission coefficient between the two ports is −22 dB, which is an acceptable limit for the ports. The *S*_13_ and *S*_14_ were also at a low value because of the larger distance between the MIMO elements. To improve the isolation level due to the small size of the circuits and antennas at 60 GHz band, we used an isolation technique (EBGs) for coupling reduction and gain improvement. Different types of the EBG structures were compared in terms of coupling reduction and gain improvement.

**Figure 7:**
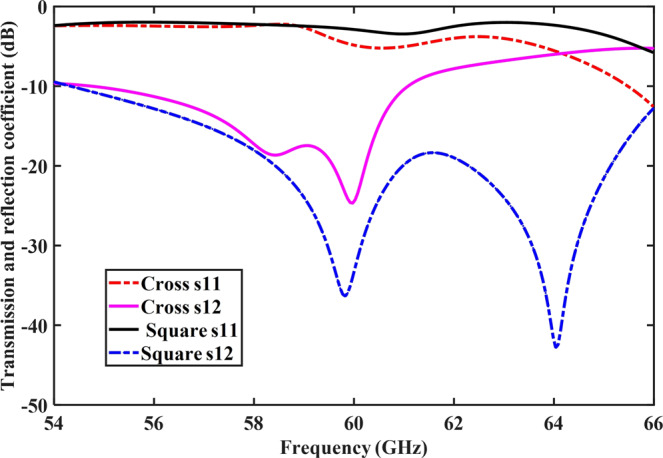
Transmission and reflection coefficients (*S*_11_, *S*_12_) of the square- and cross-shaped EBG structures. Both the EBG structures provided a wider bandgap for the surface wave suppression.

The scattering parameters of the proposed EBG structures (square- and cross-shaped) are shown in Fig. 7. It is evident from the figure that both types of EBG structures provided an acceptable bandgap at the resonant frequency. The transmission coefficients at the proposed frequency band has the lower values of −37 dB and −25 dB for the square- and cross-shaped EBG structures, respectively. The reflection coefficients have an acceptable high value of −2 dB and −5 dB for the square- and cross-shaped EBGs, respectively. These values provide broad or wide bandgap for the suppression of surface waves. The surface current distributions were shown in Fig. 4. The figure shows that the EBG structures suppress the surface waves in the desired frequency band and block them from propagation through the transmission line. The suppression capabilities of square-shaped EBG were more dominant compared to the cross-shaped EBG, where a significant number of waves were suppressed by the square-shaped EBG. The structures exhibit both the in-phase and surface-wave-suppression properties of EBG at the desired frequency band. These results proved the efficacy of the EBG structures which were then placed in-between the 60-GHz MIMO antenna elements for further analysis.

**Figure 8:**
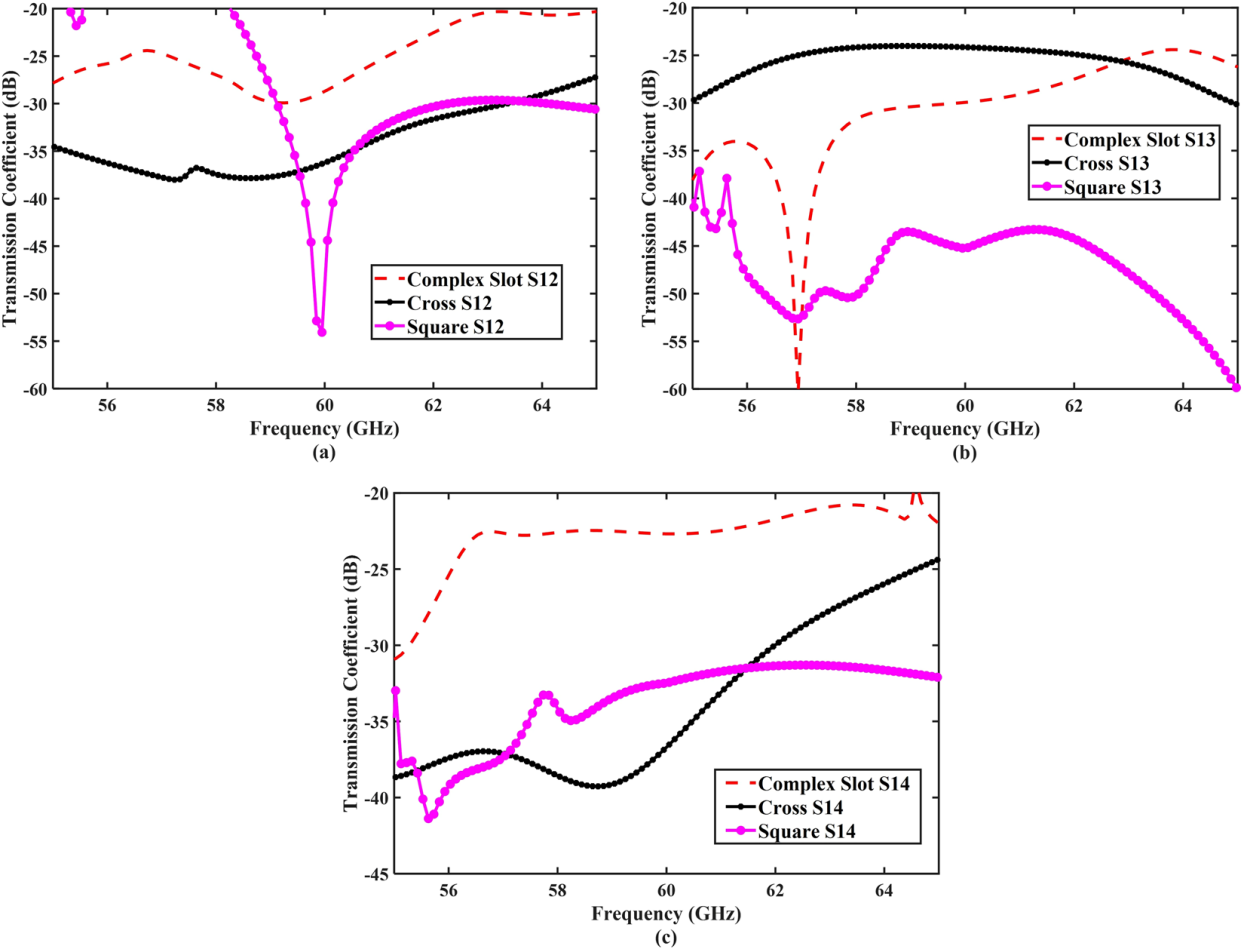
Coupling reduction of the square-shaped, cross-shaped, and complex-slotted EBG structures. (**a**) *S*_12_, (**b**) *S*_13_, (**c**) and *S*_14_. All the three EBG structures provided the coupling reduction and improved the isolation level between the array elements.

The performance of the optimized EBG structures in terms of mutual coupling reduction is shown in Fig. 8. The transmission coefficient (*S*_12_) of the MIMO antenna array with EBG structures is shown in Fig. 8(a). The mutual coupling level was −54 dB for the square-shaped EBG structure, whereas the coupling levels for the cross-shaped and complex-slotted structures were −36 dB and −30 dB, respectively. The integration of the EBG structures in the 60 GHz MIMO antenna efficiently reduced the coupling level between the adjacent elements. The square-shaped EBG structure improved the isolation from −22 dB to −54 dB and provided approximately 32 dB of coupling reduction between the antenna elements compared to the MIMO antenna without an EBG structure. The cross-shaped and complex-slotted EBG structures enhanced the isolation from −22 dB to −36 dB and −30 dB and produced 14 dB and 8 dB of coupling reduction, respectively. The investigated EBG structures suppressed the surface waves within the bandgap, resulting in a reduction in mutual coupling. Moreover, at the antenna-centered frequency, the transmission coefficient (*S*_12_) parameter showed a clear forbidden bandgap for the surface waves between the antenna elements. The scattering parameters (*S*_11_, *S*_22_, *S*_33_, *S*_44_) of the antenna elements were excellently matched at the desired 60-GHz frequency.

**Figure 9:**
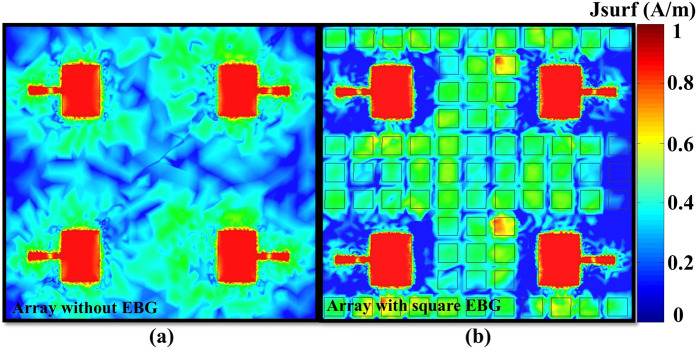
Surface currents distributions: (**a**) MIMO antenna array without EBG and (**b**) with EBG. In the absence of EBG structure, the surface currents flow toward the other elements. The integration of EBG structure isolated the MIMO array elements and reduced the current flow.

Furthermore, the scattering parameters (*S*_13_, *S*_14_) between the first element and the third and fourth elements of the array were also investigated for all the proposed types of EBG structure, as shown in Fig. 8(b,c). The isolation between the element 1 and 3 improved from −35 dB to −47 dB by using the EBG structures; however, the cross-shaped EBG structure was not that much effective in the coupling reduction of *S*_13_. Moreover, the EBG structures provided isolation between the element 1 and 4; however, owing to the large isolation gap between the elements, the EBG structures were not that much effective. To validate further the performance of the EBG structures, the surface currents flow was plotted for the MIMO antenna array with and without the square-shaped EBG, as shown in Fig. 9. As depicted from the figure, addition of the EBG structure minimized the surface currents flow into other array elements.

### Gain and radiation analysis of the MIMO antenna and the EBG structures

**Figure 10:**
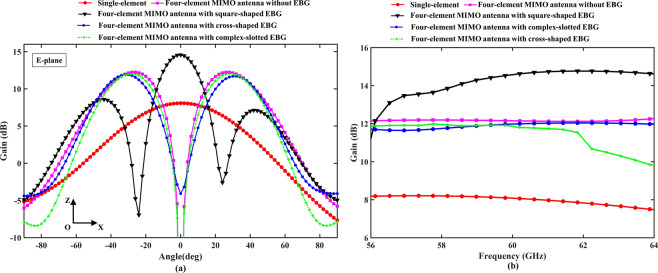
(**a**) E-plane (Azimuthal) gain comparison of the MIMO antenna array with and without EBG structures. (**b**) Gain in (dB) of one-element (without EBG structures) and four-element MIMO antenna array with and without EBG structures over the desired frequency band.

The performance of the EBG structures in terms of the gain patterns is shown against the desired direction of radiation in Fig. 10(a). The figure summarizes the gain comparison between a single-element antenna (without EBG structures) and a four-element MIMO antenna array (with and without EBG structures). It is worth noting that the EBG structures provided gain improvement, as well as coupling reduction. Satisfactorily, gain values of 8.2 dBi and 12 dBi were achieved with single-element antenna and with four-element MIMO antenna array, respectively. The square-shaped EBG structure showed significant performance enhancement as compared to that of the other EBG structures, with a wider beam-width at the desired 60-GHz frequency band. The structure provided an in-phase reflection of the surface waves and redirected them toward the maximum gain direction. An overall gain of 14.8 dBi was achieved at the desired resonant band, with 3 dBi of gain improvement. The cross-shaped and complex-slotted EBG structures provided only coupling reduction at the required 60-GHz frequency band, with no significant gain improvement; however, the structures directed the main beam slightly toward the desired direction. The small degree change in the directive beams for both EBG structures was observed in Fig. 10(a). The maximum gain over the bandwidth in the range from 56 GHz to 64 GHz for the single-element antenna (without EBG structures) and the four-element MIMO antenna array (with and without EBG structures), is shown in Fig. 10(b). The maximum gain varied between 14 dBi and 14.8 dBi over the desired bandwidth. The results reinforce the proposed argument that the simple shape EBG provides promising performance in the 60-GHz frequency band. The complex-shaped EBG structures made the size more compact, whereas the simple-shaped EBG structures performed better in the 60-GHz band.

Moreover, single-element antenna provided the maximum gain pattern in the desired direction; however, the MIMO antenna array produced two different side lobes at two distinct angles. The integration of the square-shaped EBG structure between the antenna elements provided the main lobe in the desired direction and two side lobes at two different angles. The main and two side lobes of the square-shaped EBG structure were more directive with peak gain values of 14.8 dBi and 8 dBi, respectively. All the three lobes in the desired direction can be used for the transmission and reception of the signals at mmW band for smart MIMO and high data rate applications.

### Measurement setups and results of the 60-GHz MIMO antenna array with EBG

**Figure 11:**
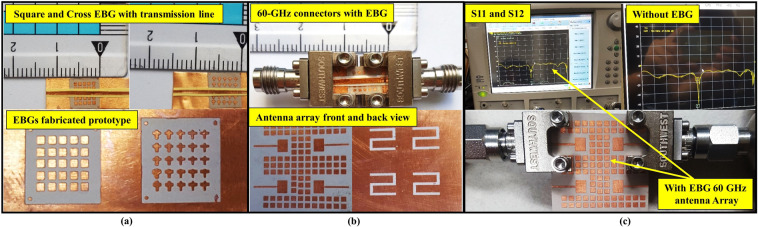
The 60-GHz antenna and measurement setups of the EBG structures. (**a**) Prototype of the fabricated EBG structures and transmission line. (**b**) 60-GHz power connectors for the input source and prototype of the square-shaped EBG with MIMO antenna array. (**c**) The 60-GHz fabricated MIMO antenna array *S*_11_ and *S*_12_ with EBG and *S*_11_ without EBG structure.

**Figure 12:**
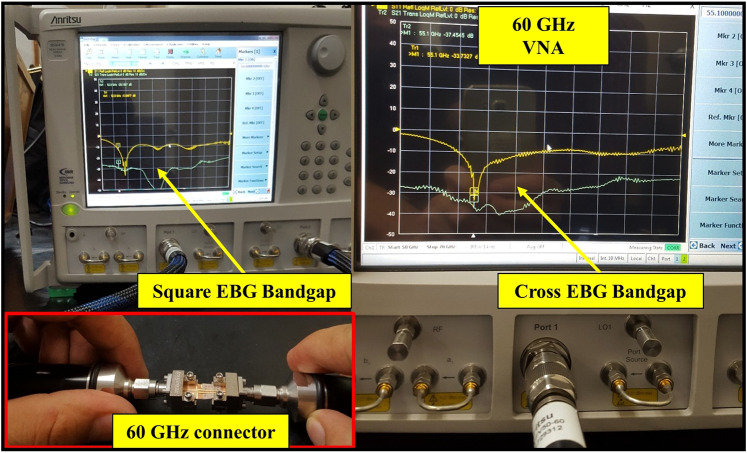
Square- and cross-shaped EBG bandgap measurement using a high-frequency (60/70 GHz) vector network analyzer (VNA). The transmission line is connected to both connectors and set to 0.3 mm above the EBG structure.

The important and complicated step in the 60-GHz band design is the fabrication and measurement of small systems. To consider the power compatibility issue of the high-frequency VNA (MS4647B Series by Anritsu) for the 60-GHz design, we connected a special type of connector (292-04A-5 SMA Female End Launch Connectors) to the proposed design. The square- and cross-shaped EBG designs were fabricated and a transmission line was placed above the structure at a distance of 0.3 mm to observe bandgap of the both structures. Both the fabricated designs and the transmission line over the EBGs with connectors are shown in Fig. 11(a,b). The two end-launch connectors were connected to the transmission line end points. The square-shaped EBG bandgap was measured using two ports of the VNA and shown in Fig. 12. The measured bandgap verified the simulation results, and at 60 GHz band −50 dB bandgap was achieved. The arrows in the figure points out the 60-GHz band and the bandgap, which starts from 56 GHz to 65 GHz. The two ports are shown connected to both connectors at the end of the transmission line. Additionally, the measured results of the cross-shaped EBG structure verified the simulation results, and the bandgap was at the center frequency (60 GHz) starting from 53 to 65 GHz with a depth below −35 dB.

Figure 11(c) shows the measurement setup for the fabricated prototype of the MIMO antenna array with and without EBG structure. Firstly, one element of the antenna array without the EBG structure was connected to the VNA and the S-parameter result was observed. Secondly, two of the MIMO antenna elements with square-shaped EBG were connected to the 60-GHz VNA through end-launch connectors. The front and backside (ground plane) views of the four elements MIMO antenna array with square-shaped EBG are shown in Fig. 11(b), while the scattering parameter results are shown in Fig. 11(c). The *S*_11_ and *S*_12_ of the antenna array with EBG structure were observed at the desired frequency band. The results were slightly shifted owing to the connector losses and fabrication errors. The center frequency was at 59 GHz, although the antenna still adequately covered the 60-GHz band.

**Figure 13:**
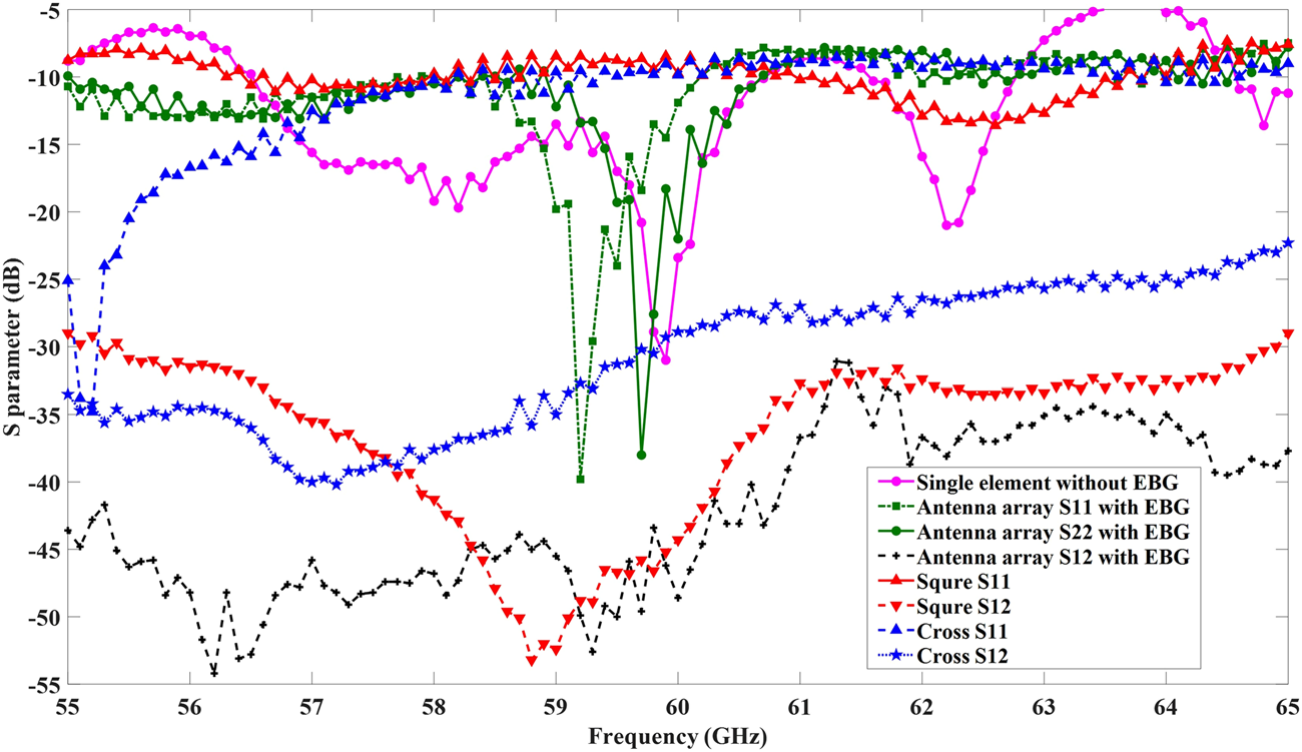
Measured reflection coefficients (S-parameter) for the two EBG structures and the 60-GHz antenna array *S*_11_ and *S*_12_ with and without EBG structures. The measured *S*_11_, *S*_22_, and *S*_12_ for MIMO antenna array with EBG structure, *S*_11_ and *S*_12_ for both EBG structures (square- and cross-shaped), and the *S*_11_ coefficient for the 60-GHz one-element antenna without the EBG structures are shown.

The single-element of the MIMO antenna array with EBG structure was measured and it had a return loss below −40 dB at 59.5 GHz. The *S*_11_ and *S*_22_ parameters were shifted slightly from the central frequency due to the fabrication errors of the EBG unit cells near the antenna elements. The measured *S*_11_ and *S*_12_ for both EBGs (square- and cross-shaped) and the *S*_11_, *S*_22_, and *S*_12_ coefficients for the 60-GHz antenna array with and without EBG structures are shown in Fig. 13. The measured bandgap of the EBG structures worked in the mutual coupling reduction and gain improvement of the MIMO antenna array. The square-shaped EBG structure reduced the *S*_12_ coefficient, and the value was improved to −52 dB at the resonant frequency. The reduction factor in the mutual coupling was significant. The figure shows that the measurement results were in a reasonable agreement with the simulation results, whereas slight shifts were observed in the results owing to the connector losses and fabrication errors.

### Radiation patterns Measurement of the 60-GHz antenna with EBG

**Figure 14:**
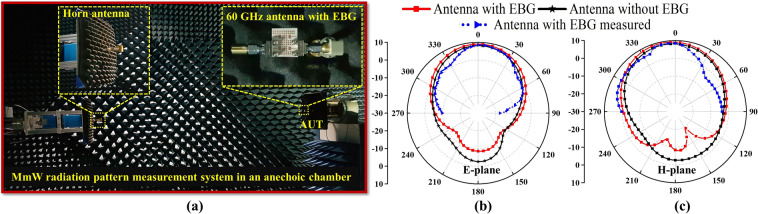
Measured and simulated radiation patterns of a 60 GHz antenna. (**a**) Measurement setup. (**b**) E-plane (azimuthal). (**c**) H-plane (elevation).

Figure 14(a) demonstrates the measurement setup for the radiation pattern of a 60-GHz antenna with EBG in a commercial ORBIT/FR far-field anechoic chamber. The far-field measurements were performed in the E- and H-planes with theta range of −90° to 90° to understand the radiation behavior of our proposed 60 GHz antenna. In Fig. 14(b,c) the simulated and measured radiation patterns are shown at 60 GHz for the single element of the antenna. As can be seen from the figure, the gain patterns of simulated and measured data follow nearly the same patterns. The discrepancy between the simulated and measured data is due to the fabrication errors and measurement setup which is not most appropriate for small antennas. Moreover, it was observed that the back lobes were minimized, and the peak gain was increased by loading the EBG structure. The peak gains of 8.2, 9.1, and 8.7 dBi were observed for the single-element simulated without EBG, simulated with EBG, and measured with EBG, respectively, at the desired frequency of 60 GHz. The measured gain values and its patterns agreed well with simulated ones.

### Effects of the gap between unit cells and distance between the antenna elements and EBG

In the EBG optimization, the length and gap between unit cells were investigated using different dimensions. At the gap of 0.3 mm and 0.09 mm, the EBG provided an acceptable bandgap for the coupling reduction and gain improvement. Therefore, these gaps between the unit cells were investigated for the square- and cross-shaped EBG structures. For the square-shaped EBG structure, the unit cells gap of 0.3 mm produced an acceptable coupling reduction and gain improvement in the desired frequency band, whereas the cross-shaped EBG structure provided an acceptable coupling reduction for the 0.09 mm gap width. The results show that the simple shaped EBG provided better performance with ease in the fabrication of EBG structure, whereas the cross-shaped and complex-slotted EBGs provided almost similar results by using very small size of the EBG that produced complexity in fabrication and required costly equipment. In addition, the effects of distance variation between the antenna elements and EBG were investigated. From the simulation results, we observed that the square-shaped EBG performed well and produced a small variation in the *S*_11_ compared to the cross- and complex-shaped EBGs. At 60 GHz, the wavelength becomes very small, and the multiple shape (complex shapes) variation in the EBG structure disturbs the EBG current distributions. Therefore, the square-shaped EBG performed better at 60-GHz band and provided ease in the design and fabrication process.

In this work, we used different types of EBG structures for comparison such as square-shaped and two complex shapes to investigate the effects of shape on the performance at the 60-GHz band. The complex shapes EBG structures miniaturized the size of the EBG ground plane at the lower frequency bands (2.4 GHz)^30^. At lower frequency band, the compact and complex shapes EBG produced better performance compared to the simple shapes, whereas at higher frequencies like 60-GHz the simple shapes EBGs were more promising. Our EBG comparison based on coupling reduction, gain, and radiation pattern shows that the square-shaped EBG provides promising results compared to the cross and complex EBG shapes. A miniaturized four-element MIMO antenna with EBG structure was suggested for Implantable medical devices^31^. The proposed design can also be implemented in the near future for the body surface imaging at 60 GHz. Thus, this study suggests the simple shape EBG structure for the higher frequency bands (60-GHz).

## Conclusion

This study compared and experimentally validated different types of EBG structures in a grounded multi-element MIMO antenna array. The EBG structures reduced the mutual coupling and improved the gain of the MIMO antenna array. The proposed MIMO antenna with EBG structures at 60 GHz can be mounted on a chip, and it can also act as a smart antenna array for MIMO applications in transmission/reception systems. We used a HFSS simulator for modeling and optimization of the array. For the surface wave suppression, we investigated the reflection and transmission coefficients of the square- and cross-shaped EBG structures, while a wide bandgap at our desired bandwidth was observed. To achieve the mutual coupling reduction and gain improvement of the MIMO antenna array at desired frequency band, we placed the square-shaped, cross-shaped, and complex-slotted structures of the EBG between the elements. The square-shaped EBG structure provided an approximately 3 dB gain improvement, while the mutual coupling reduction of 32 dB, 14 dB, and 8 dB were observed for the square-shaped, cross-shaped, and complex-slotted EBG structures, respectively. The EBG structures and antenna elements were fabricated to verify the simulation results. The measurement results showed a reasonable agreement with the simulation, while the square-shaped EBG produced better performance compared to the cross-shaped EBG structure. The proposed MIMO antenna array with square-shaped EBG structure has the potential to be used for a MIMO communication with high data rates.
